# The genome sequence of the common toadflax, *Linaria vulgaris* Mill., 1768

**DOI:** 10.12688/wellcomeopenres.19661.1

**Published:** 2023-08-30

**Authors:** Maarten J. M. Christenhusz, Benjamin Fisk, Meng Lu, Royal Botanic Gardens Kew Genome Acquisition Lab, Royal Botanic Garden Edinburgh Genome Acquisition Lab, Plant Genome Sizing collective, Darwin Tree of Life Barcoding collective, Wellcome Sanger Institute Tree of Life programme, Wellcome Sanger Institute Scientific Operations: DNA Pipelines collective, Tree of Life Core Informatics collective, Darwin Tree of Life Consortium

**Affiliations:** 1 Royal Botanic Gardens Kew, Richmond, England, UK; 2 University of Cambridge, Cambridge, England, UK; 3 The University of Edinburgh, Edinburgh, Scotland, UK; 4 Royal Botanic Garden Edinburgh, Edinburgh, Scotland, UK

**Keywords:** Linaria vulgaris, common toadflax, genome sequence, chromosomal, Lamiales

## Abstract

We present a genome assembly from a *Linaria vulgaris* specimen (common toadflax; Streptophyta; Magnoliopsida; Lamiales; Plantaginaceae). The genome sequence is 760.5 megabases in span. Most of the assembly is scaffolded into six chromosomal pseudomolecules. Two mitochondrial genomes were assembled, which were 330.8 and 144.0 kilobases long. The plastid genome was also assembled and is 156.7 kilobases in length.

## Species taxonomy

Eukaryota; Viridiplantae; Streptophyta; Embryophyta; Tracheophyta; Spermatophyta; Magnoliopsida; eudicotyledons; Gunneridae; Pentapetalae; asterids; lamiids; Lamiales; Plantaginaceae; Antirrhineae; *Linaria*; *Linaria vulgaris* Mill. (NCBI:txid43171).

## Background

Common toadflax, *Linaria vulgaris* Mill. (Plantaginaceae; 2 *n* = 2 *x* = 12 ( Henniges *et al.*, 2022), is a short-lived herbaceous perennial plant. It is native to most of Europe to North and Central Asia, the Himalayas and eastern China ( POWO, 2022), and was introduced to South Africa, northeast Asia and the temperate Americas, where it can be highly invasive ( Sing & Peterson, 2011). It is widely distributed throughout Britain and Ireland, although it is less common in northern Scotland and western Ireland ( Preston *et al.*, 2002; Stace *et al.*, 2019). *Linaria vulgaris* avoids acid soils but tolerates a wide range of habitats, such as open meadows and disturbed roadsides ( Sing & Peterson, 2011; Stace *et al.*, 2019). This species readily hybridises with other members of its genus ( Stace *et al.*, 2015; Ward *et al.*, 2009). One of the best-studied *Linaria* hybrids in Britain and Ireland is *L. vulgaris* × *repens*, which is highly fertile and able to backcross with both parental species ( Druce, 1896; Stace *et al.*, 2015).

**Figure 1.**
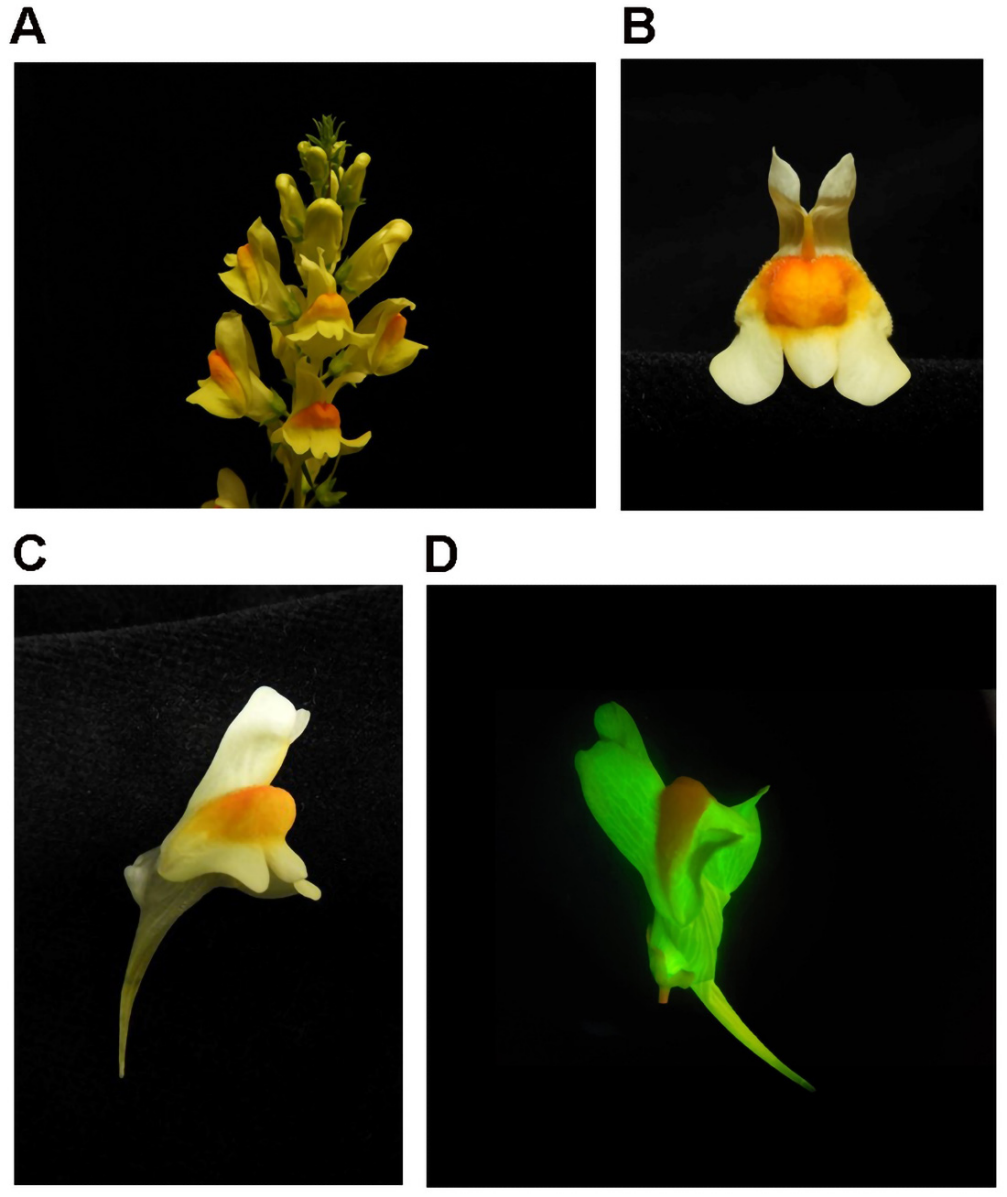
Flowers of *Linaria vulgaris* (not the sampled specimen). **A**. Flowers are borne on terminal racemes. **B**. Flowers are lobed and bilaterally symmetrical. **C**. Each flower possesses a single nectar spur. **D** Photograph of *Linaria vulgaris* expressing YFP following Agrobacterium-mediated transformation.

The foliage superficially resembles that of the flax ( *Linum L.)*, whence the names ‘toadflax’ and ‘ *Linaria*’ derive ( Gledhill, 2008). The zygomorphic flowers, borne on terminal racemes, are pale-yellow with orange centres ( Figure 1A, B). Each flower contains a tubular nectar spur, which holds a reservoir of nectar that is accessible to long-tongued bumblebee pollinators ( Newman & Thomson, 2005) ( Figure 1C). *Linaria vulgaris* reproduces sexually, via bee-mediated pollination, and asexually, via vegetative propagation from adventitious buds which develop on the roots ( Bakshi & Coupland, 1960). Seeds are dust-like and dispersed by wind.

*Linaria vulgaris* has been of particular interest to botanists for over 250 years, since Linnaeus described mutant toadflax flowers that displayed radial, instead of the typical bilateral, symmetry ( Gustafsson, 1979; Linneaus, 1749). Later characterisation of these mutant flowers led to the first description of naturally occurring epigenetic mutations (epimutations) ( Cubas *et al.*, 1999). It is currently being developed as a model system for studying nectar spur development and floral evolution ( Cullen *et al.*, 2018), and has recently been genetically transformed via Agrobacterium-mediated transformation ( Figure 1D).

We hope that the sequence provided here will contribute to the study of *Linaria vulgaris* as an emerging model for understanding the genetics of floral evolution and flower development. This whole genome sequence will also facilitate population genetic studies of natural toadflax populations.

## Genome sequence report

The genome was sequenced from a specimen of *Linaria vulgaris* ( Figure 1) collected from along the River Thames towpath in Kingston upon Thames, Surrey, UK (latitude 51.43, longitude –0.31). Using flow cytometry, the genome size (1C-value) was estimated to be 0.96 pg, equivalent to 940 Mb. A total of 28-fold coverage in Pacific Biosciences single-molecule HiFi long reads was generated. Primary assembly contigs were scaffolded with chromosome conformation Hi-C data. Manual assembly curation corrected 69 missing joins or mis-joins and removed 58 haplotypic duplications, reducing the assembly length by 3.2% and the scaffold number by 59.8%, and decreasing the scaffold N50 by 17.75%.

**Figure 2.**
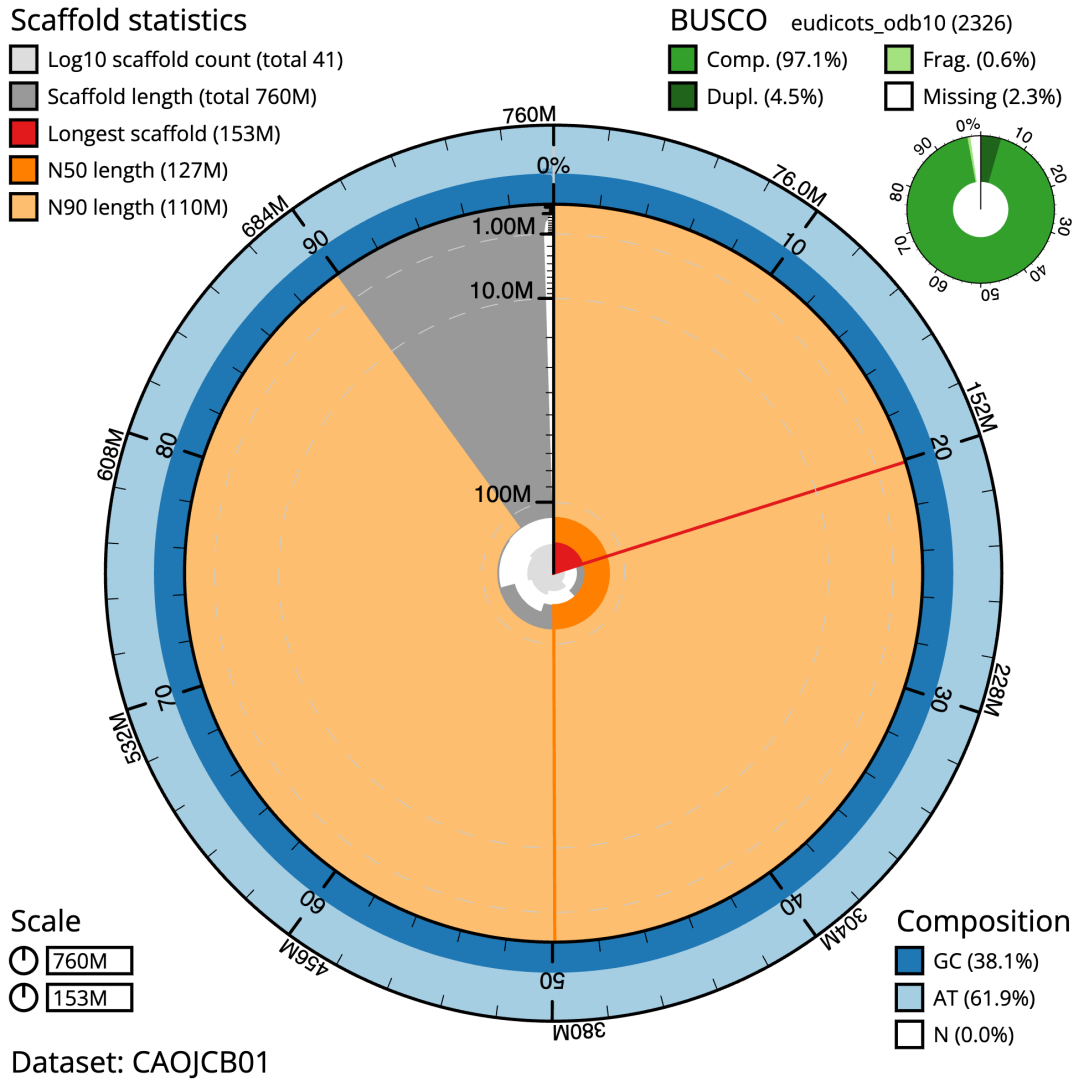
Genome assembly of *Linaria vulgaris*, daLinVulg1.1: metrics. The BlobToolKit Snailplot shows N50 metrics and BUSCO gene completeness. The main plot is divided into 1,000 size-ordered bins around the circumference with each bin representing 0.1% of the 760,456,579 bp assembly. The distribution of scaffold lengths is shown in dark grey with the plot radius scaled to the longest scaffold present in the assembly (153,338,251 bp, shown in red). Orange and pale-orange arcs show the N50 and N90 scaffold lengths (127,462,361 and 110,092,459 bp), respectively. The pale grey spiral shows the cumulative scaffold count on a log scale with white scale lines showing successive orders of magnitude. The blue and pale-blue area around the outside of the plot shows the distribution of GC, AT and N percentages in the same bins as the inner plot. A summary of complete, fragmented, duplicated and missing BUSCO genes in the eudicots_odb10 set is shown in the top right. An interactive version of this figure is available at https://blobtoolkit.genomehubs.org/view/daLinVulg1.1/dataset/CAOJCB01/snail.

The final assembly has a total length of 760.5 Mb in 41 sequence scaffolds with a scaffold N50 of 127.5 Mb ( Table 1). Most (99.62%) of the assembly sequence was assigned to six chromosomal-level scaffolds. Chromosome-scale scaffolds confirmed by the Hi-C data are named in order of size ( Figure 2– Figure 5; Table 2). While not fully phased, the assembly deposited is of one haplotype. Contigs corresponding to the second haplotype have also been deposited.

**Table 1.** Genome data for *Linaria vulgaris*, daLinVulg1.1.

Project accession data		
Assembly identifier	daLinVulg1.1	
Species	*Linaria vulgaris*	
Specimen	daLinVulg1	
NCBI taxonomy ID	43171	
BioProject	PRJEB50878	
BioSample ID	SAMEA7522288	
Isolate information	daLinVulg1; leaf (genome sequencing and Hi-C scaffolding); flower (RNA sequencing)	
Assembly metrics *		*Benchmark*
Consensus quality (QV)	61.8	*≥ 50*
*k*-mer completeness	100%	*≥ 95%*
BUSCO **	C:97.1%[S:92.6%,D:4.5%], F:0.6%,M:2.3%,n:2,326	*C ≥ 95%*
Percentage of assembly mapped to chromosomes	99.62%	*≥ 95%*
Sex chromosomes	Not applicable	*localised homologous pairs*
Organelles	Two mitochondrial scaffolds, one chloroplast scaffold	*complete single alleles*
Raw data accessions		
PacificBiosciences SEQUEL II	ERR8705859, ERR8705860	
Hi-C Illumina	ERR8702786	
PolyA RNA-Seq Illumina	ERR10378004, ERR10378005	
Genome assembly		
Assembly accession	GCA_948329865.1	
*Accession of alternate haplotype*	GCA_948329855.1	
Span (Mb)	760.5	
Number of contigs	132	
Contig N50 length (Mb)	12.7	
Number of scaffolds	41	
Scaffold N50 length (Mb)	127.5	
Longest scaffold (Mb)	153.3	

* Assembly metric benchmarks are adapted from column VGP-2020 of “Table 1: Proposed standards and metrics for defining genome assembly quality” from ( Rhie *et al.*, 2021). ** BUSCO scores based on the eudicots_odb10 BUSCO set using v5.3.2. C = complete [S = single copy, D = duplicated], F = fragmented, M = missing, n = number of orthologues in comparison. A full set of BUSCO scores is available at https://blobtoolkit.genomehubs.org/view/daLinVulg1.1/dataset/CAOJCB01/busco.

**Figure 3.**
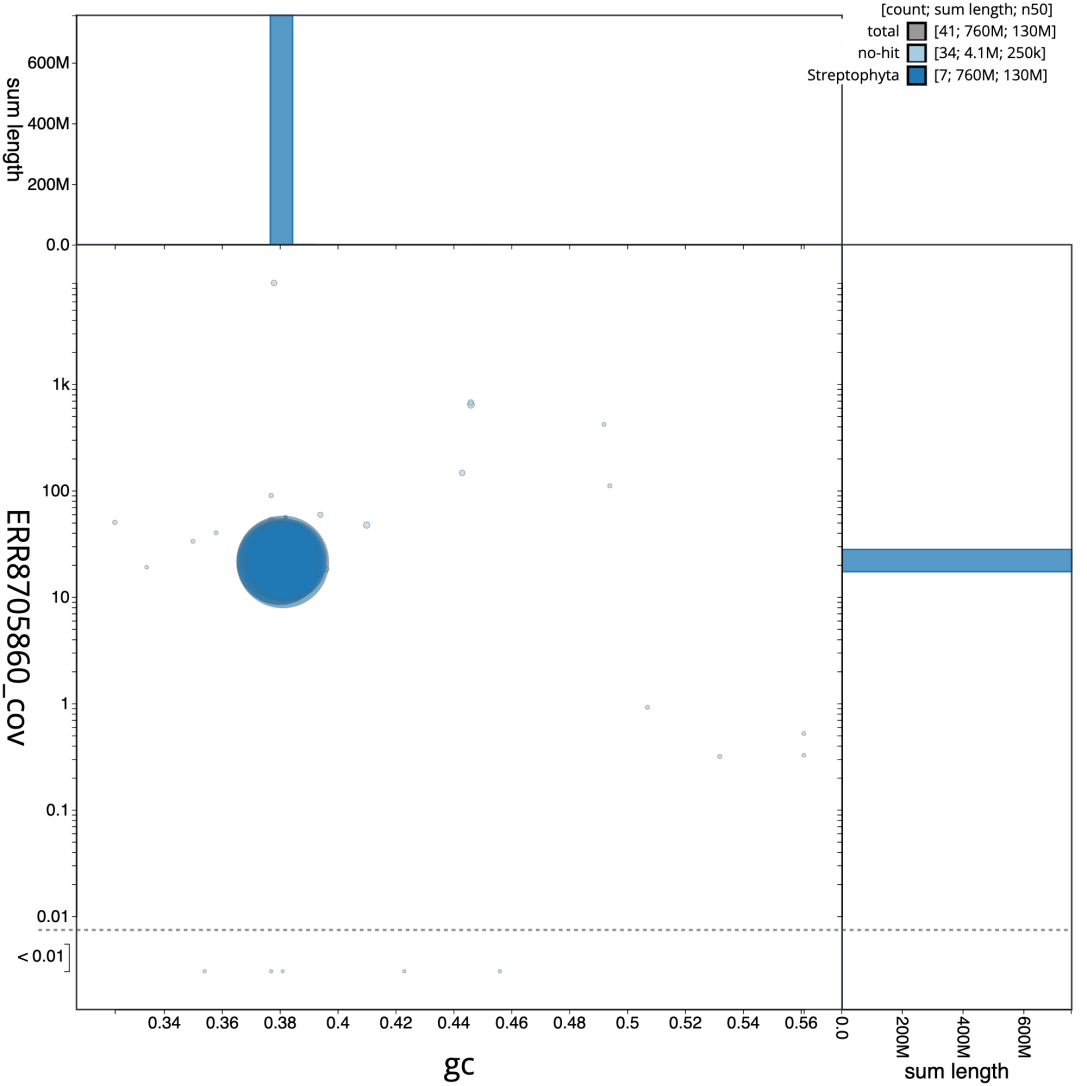
Genome assembly of *Linaria vulgaris*, daLinVulg1.1: GC coverage. BlobToolKit GC-coverage plot. Scaffolds are coloured by phylum. Circles are sized in proportion to scaffold length. Histograms show the distribution of scaffold length sum along each axis. An interactive version of this figure is available at https://blobtoolkit.genomehubs.org/view/daLinVulg1.1/dataset/CAOJCB01/blob.

**Figure 4.**
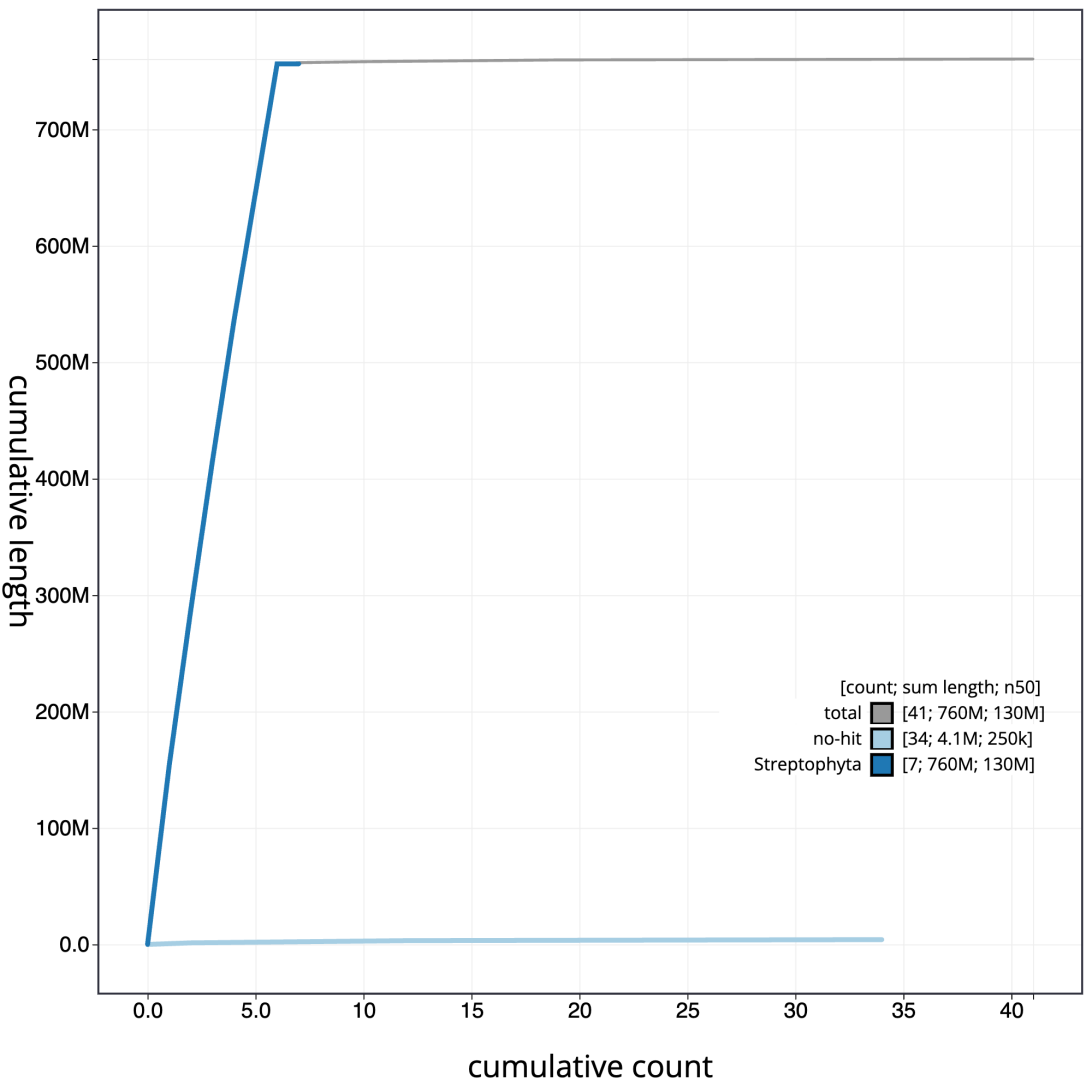
Genome assembly of *Linaria vulgaris*, daLinVulg1.1: cumulative sequence. BlobToolKit cumulative sequence plot. The grey line shows cumulative length for all scaffolds. Coloured lines show cumulative lengths of scaffolds assigned to each phylum using the buscogenes taxrule. An interactive version of this figure is available at https://blobtoolkit.genomehubs.org/view/daLinVulg1.1/dataset/CAOJCB01/cumulative.

**Figure 5.**
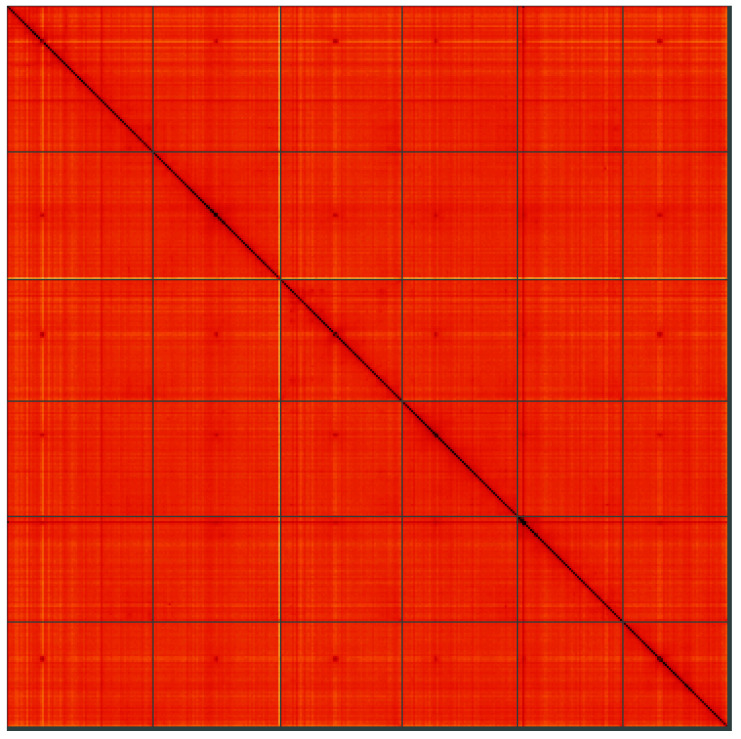
Genome assembly of *Linaria vulgaris*, daLinVulg1.1: Hi-C contact map. Hi-C contact map of the daLinVulg1.1 assembly, visualised using HiGlass. Chromosomes are shown in order of size from left to right and top to bottom. An interactive version of this figure may be viewed at https://genome-note-higlass.tol.sanger.ac.uk/l/?d=Lroe3SmHRWOiVBKAhWt8bQ.

**Table 2.** Chromosomal pseudomolecules in the genome assembly of *Linaria vulgaris*, daLinVulg1.

INSDC accession	Chromosome	Size (Mb)	GC%
OX415249.1	1	153.34	38.1
OX415250.1	2	133.81	38
OX415251.1	3	127.46	38
OX415252.1	4	120.92	38.2
OX415253.1	5	110.69	38
OX415254.1	6	110.09	38.2
OX415255.1	MT1	0.33	44.5
OX415256.1	MT2	0.14	44.6
OX415257.1	Pltd	0.16	37.8
-	unplaced	3.51	39.6

The estimated Quality Value (QV) of the final assembly is 61.8 with *k*-mer completeness of 100%, and the assembly has a BUSCO v5.3.2 completeness of 97.1% (single = 92.6%, duplicated = 4.5%), using the eudicots_odb10 reference set ( *n* = 2,326).

Metadata for specimens, spectral estimates, sequencing runs, contaminants and pre-curation assembly statistics can be found at https://links.tol.sanger.ac.uk/species/43171.

## Methods

### Sample acquisition, genome size estimation and nucleic acid extraction

A *Linaria vulgaris* specimen (daLinVulg1) was collected from Kingston upon Thames, Surrey, UK (latitude 51.43, longitude –0.31) on 1 September 2020. The specimen was picked by hand by Maarten Christenhusz (Royal Botanic Gardens, Kew) from riparian vegetation on sand along the River Thames towpath. The specimen was identified based on its morphology by Maarten Christenhusz, and was preserved by freezing at –80°C.

The genome size was estimated by flow cytometry using the fluorochrome propidium iodide and following the ‘one-step’ method as outlined in Pellicer *et al*. (2021). Specifically for this species, the General Purpose Buffer (GPB) supplemented with 3% PVP and 0.08% (v/v) beta-mercaptoethanol was used for isolation of nuclei ( Loureiro *et al.*, 2007), and the internal calibration standard was *Petroselinum crispum* ‘Champion Moss Curled’ with an assumed 1C-value of 2,200 Mb ( Obermayer *et al.*, 2002).

DNA was extracted at the Tree of Life laboratory, Wellcome Sanger Institute (WSI). The daLinVulg1 sample was weighed and dissected on dry ice with tissue set aside for Hi-C sequencing. Leaf tissue was cryogenically disrupted to a fine powder using a Covaris cryoPREP Automated Dry Pulveriser, receiving multiple impacts. High molecular weight (HMW) DNA was extracted using the Illustra Nucleon PhytoPure HMW DNA extraction kit. HMW DNA was sheared into an average fragment size of 12–20 kb in a Megaruptor 3 system with speed setting 30. Sheared DNA was purified by solid-phase reversible immobilisation using AMPure PB beads with a 1.8× ratio of beads to sample to remove the shorter fragments and concentrate the DNA sample. The concentration of the sheared and purified DNA was assessed using a Nanodrop spectrophotometer and Qubit Fluorometer and Qubit dsDNA High Sensitivity Assay kit. Fragment size distribution was evaluated by running the sample on the FemtoPulse system.

RNA was extracted from flower tissue of daLinVulg1 in the Tree of Life Laboratory at the WSI using TRIzol, according to the manufacturer’s instructions. RNA was then eluted in 50 μl RNAse-free water and its concentration assessed using a Nanodrop spectrophotometer and Qubit Fluorometer using the Qubit RNA Broad-Range (BR) Assay kit. Analysis of the integrity of the RNA was done using the Agilent RNA 6000 Pico Kit and Eukaryotic Total RNA assay.

### Sequencing

Pacific Biosciences HiFi circular consensus DNA sequencing libraries were constructed according to the manufacturers’ instructions. Poly(A) RNA-Seq libraries were constructed using the NEB Ultra II RNA Library Prep kit. DNA and RNA sequencing was performed by the Scientific Operations core at the WSI on Pacific Biosciences SEQUEL II (HiFi) and Illumina NovaSeq 6000 (RNA-Seq) instruments. Hi-C data were also generated from leaf tissue of daLinVulg1 using the Arima2 kit and sequenced on the Illumina NovaSeq 6000 instrument.

### Genome assembly, curation and evaluation

Assembly was carried out with Hifiasm ( Cheng *et al.*, 2021) and haplotypic duplication was identified and removed with purge_dups ( Guan *et al.*, 2020). One round of polishing was performed by aligning 10X Genomics read data to the assembly with Long Ranger ALIGN, calling variants with FreeBayes ( Garrison & Marth, 2012). The assembly was then scaffolded with Hi-C data ( Rao *et al.*, 2014) using YaHS ( Zhou *et al.*, 2023). The assembly was checked for contamination and corrected using the gEVAL system ( Chow *et al.*, 2016) as described previously ( Howe *et al.*, 2021). Manual curation was performed using gEVAL, HiGlass ( Kerpedjiev *et al.*, 2018) and Pretext ( Harry, 2022). The mitochondrial and chloroplast genomes were assembled using MBG ( Rautiainen & Marschall, 2021) from PacBio HiFi reads mapping to related genomes: a representative circular sequence was selected for each from the graph based on read coverage.

A Hi-C map for the final assembly was produced using bwa-mem2 ( Vasimuddin *et al.*, 2019) in the Cooler file format ( Abdennur & Mirny, 2020). To assess the assembly metrics, the k-mer completeness and QV consensus quality values were calculated in Merqury ( Rhie *et al.*, 2020). This work was done using Nextflow ( Di Tommaso *et al.*, 2017) DSL2 pipelines “sanger-tol/readmapping” ( Surana *et al.*, 2023a) and “sanger-tol/genomenote” ( Surana *et al.*, 2023b). The genome was analysed within the BlobToolKit environment ( Challis *et al.*, 2020) and BUSCO scores ( Manni *et al.*, 2021; Simão *et al.*, 2015) were calculated.

Table 3 contains a list of relevant software tool versions and sources.

**Table 3.** Software tools: versions and sources.

Software tool	Version	Source
BlobToolKit	4.0.7	https://github.com/blobtoolkit/blobtoolkit
BUSCO	5.3.2	https://gitlab.com/ezlab/busco
FreeBayes	1.3.1-17-gaa2ace8	https://github.com/freebayes/freebayes
gEVAL	N/A	https://geval.org.uk/
Hifiasm	0.15.3	https://github.com/chhylp123/hifiasm
HiGlass	1.11.6	https://github.com/higlass/higlass
Long Ranger ALIGN	2.2.2	https://support.10xgenomics.com/genome- exome/software/pipelines/latest/advanced/other- pipelines
MBG	-	https://github.com/maickrau/MBG
Merqury	MerquryFK	https://github.com/thegenemyers/MERQURY.FK
PretextView	0.2	https://github.com/wtsi-hpag/PretextView
purge_dups	1.2.3	https://github.com/dfguan/purge_dups
YaHS	yahs-1.1.91eebc2	https://github.com/c-zhou/yahs

### Wellcome Sanger Institute – Legal and Governance

The materials that have contributed to this genome note have been supplied by a Darwin Tree of Life Partner. The submission of materials by a Darwin Tree of Life Partner is subject to the **‘Darwin Tree of Life Project Sampling Code of Practice’**, which can be found in full on the Darwin Tree of Life website here. By agreeing with and signing up to the Sampling Code of Practice, the Darwin Tree of Life Partner agrees they will meet the legal and ethical requirements and standards set out within this document in respect of all samples acquired for, and supplied to, the Darwin Tree of Life Project. 

Further, the Wellcome Sanger Institute employs a process whereby due diligence is carried out proportionate to the nature of the materials themselves, and the circumstances under which they have been/are to be collected and provided for use. The purpose of this is to address and mitigate any potential legal and/or ethical implications of receipt and use of the materials as part of the research project, and to ensure that in doing so we align with best practice wherever possible. The overarching areas of consideration are:

•   Ethical review of provenance and sourcing of the material

•   Legality of collection, transfer and use (national and international)

Each transfer of samples is further undertaken according to a Research Collaboration Agreement or Material Transfer Agreement entered into by the Darwin Tree of Life Partner, Genome Research Limited (operating as the Wellcome Sanger Institute), and in some circumstances other Darwin Tree of Life collaborators.

## Data Availability

European Nucleotide Archive: *Linaria vulgaris*. Accession number PRJEB50878; https://identifiers.org/ena.embl/PRJEB50878 ( Wellcome Sanger Institute, 2022) The genome sequence is released openly for reuse. The *Linaria vulgaris* genome sequencing initiative is part of the Darwin Tree of Life (DToL) project. All raw sequence data and the assembly have been deposited in INSDC databases. The genome will be annotated using available RNA-Seq data and presented through the Ensembl pipeline at the European Bioinformatics Institute. Raw data and assembly accession identifiers are reported in Table 1.

## Author information

Members of the Royal Botanic Gardens Kew Genome Acquisition Lab are listed here: https://doi.org/10.5281/zenodo.4786680.

Members of the Royal Botanic Garden Edinburgh Genome Acquisition Lab are listed here: https://doi.org/10.5281/zenodo.4786682.

Members of the Plant Genome Sizing collective are listed here: https://doi.org/10.5281/zenodo.7994306.

Members of the Darwin Tree of Life Barcoding collective are listed here: https://doi.org/10.5281/zenodo.4893703.

Members of the Wellcome Sanger Institute Tree of Life programme are listed here: https://doi.org/10.5281/zenodo.4783585.

Members of Wellcome Sanger Institute Scientific Operations: DNA Pipelines collective are listed here: https://doi.org/10.5281/zenodo.4790455.

Members of the Tree of Life Core Informatics collective are listed here: https://doi.org/10.5281/zenodo.5013541.

Members of the Darwin Tree of Life Consortium are listed here: https://doi.org/10.5281/zenodo.4783558.
