# Supplementary material for: The genome sequence of the common toadflax, Linaria vulgaris Mill., 1768
Source: Wellcome Open Res. 2023 Aug 30;8:370. [Version 1] doi: 10.12688/wellcomeopenres.19661.1 (PMC11384191; doi:10.12688/wellcomeopenres.19661.1)
Supplement: Peer review history

# Reviewer report: Reviewer response for version 1

**DOI:** 10.21956/wellcomeopenres.21778.r95003

**Published:** 2024-09-09

**Authors:** Kai-Hua Jia

**Affiliations:** 1 Shandong Academy of Agricultural Sciences, Jinan, China

The authors present a high-quality genome assembly of *Linaria vulgaris* (common toadflax), providing valuable insights into the species' genetics and evolutionary biology. This genome, assembled with a combination of PacBio HiFi, Illumina, and Hi-C sequencing data, represents a significant advancement in the understanding of this species. The assembly of both mitochondrial and plastid genomes, along with chromosomal-level pseudomolecules, adds substantial value to this work, especially for population genetics and evolutionary studies. The manuscript is well-written, and the methods are clearly described.

Are sufficient details of methods and materials provided to allow replication by others?

Yes

Is the rationale for creating the dataset(s) clearly described?

Yes

Are the datasets clearly presented in a useable and accessible format?

Yes

Are the protocols appropriate and is the work technically sound?

Yes

Reviewer Expertise:

genome

I confirm that I have read this submission and believe that I have an appropriate level of expertise to confirm that it is of an acceptable scientific standard.

# Reviewer report: Reviewer response for version 1

**DOI:** 10.21956/wellcomeopenres.21778.r66308

**Published:** 2023-09-21

**Authors:** Xin Liu

**Affiliations:** 1 State Key Laboratory of Agricultural Genomics, BGI (Beijing Genomics Institute)-Shenzhen, Shenzhen, China

The data note by Maarten et al. described the established dataset of the common toadflax genome. The dataset is of good quality, with background information and methods well described. As a data note, I think it is acceptable and can be further used in future studies. 

 Meanwhile, I have two suggestions, in which I would recommend the authors to provide further information. First of all, the assembled genome size was substantially smaller than the estimated genome size (by flow cytometry). It would be better if the authors can provide further information (for example, estimation through kmer analysis), or maybe discussion on possible reason for the inconsistency here. Secondly, although I do not know whether it is required for the DToL project, I would suggest including gene annotation result in the dataset, which would make the dataset to be more complete and reusable.

Are sufficient details of methods and materials provided to allow replication by others?

Yes

Is the rationale for creating the dataset(s) clearly described?

Yes

Are the datasets clearly presented in a useable and accessible format?

Yes

Are the protocols appropriate and is the work technically sound?

Yes

Reviewer Expertise:

genomics, plant genomics, bioinformatics

I confirm that I have read this submission and believe that I have an appropriate level of expertise to confirm that it is of an acceptable scientific standard.
